# Participant versus informant reports of changes in social, emotional, and financial functioning after an exercise intervention with older adults: an actor-partner interdependence model analysis

**DOI:** 10.3389/fpsyg.2026.1823279

**Published:** 2026-06-02

**Authors:** Kyle A. Chrystal, Margy Y. Chen, Angela D. Bryan, Carillon J. Skrzynski

**Affiliations:** Department of Psychology and Neuroscience, University of Colorado Boulder, Boulder, CO, United States

**Keywords:** daily functioning, dyad, financial capacity, life satisfaction, older adults

## Abstract

Informant reports are potentially valuable, yet underutilized, measures for non-cognitive outcomes in older adults. Specifically, informant reports from close others may offer insights into changes in observable functional status and behavior beyond what participants report. In the context of an experimental exercise protocol in older adults, informant reports could potentially demonstrate changes in functional status and behavior that are unnoticed by participants but observable by others, based on the reliability of some informant report measures in this population. Given the interdependence of self-report and informant-report measures of a participant’s behavior, this study aimed to investigate the association between self-report and informant-report measures across an experimental exercise protocol by utilizing an Actor-Partner Interdependence Model (APIM) approach to concurrently analyze the report styles. A total of 273 participants (61.17% Female, 91.57% White, *M*_age_ = 67.92) and 117 informants (primarily spouses and/or family members) participated in the study. Participants completed a 16-week exercise program. Participants were asked to report on their life satisfaction, financial capacity, and problems with daily functioning at baseline and the end of the program, while informants were asked to report on their interpretation of the participant’s life satisfaction, financial capacity, and problems with daily functioning at the same timepoints. APIMs showed that higher informant-reported baseline life satisfaction was associated with higher participant self-reported life satisfaction following the 16-week experimental protocol. In contrast, there was divergence in self-reported and informant-reported financial capacity. Higher baseline informant reports were associated with lower informant follow-up reports but higher participant follow-up reports. Finally, daily functioning was more aligned. Participants saw an overall decrease in problems with daily functioning from baseline to follow-up, and higher participant baseline reports of problems with daily functioning were associated with lower follow-up reports in both participants and informants. The analysis shows that the APIM effectively captures concordance and discrepancy between report styles and suggests APIM may be an effective tool to most accurately interpret outcomes in older adults when both self-report and informant-report measures are collected.

## Introduction

Given the utility and importance of behavioral and cognitive outcomes in older adults (Chodosh et al., 2004; Dillon et al., 2013), psychology researchers have long aimed to introduce more reliable behavioral and cognitive measures. In studies related to changes in functioning over time, researchers have primarily relied on self-report measures for insight into behavior and cognition. However, self-report measures may not always be the most reliable method for certain constructs (Paulhus, 1991; Brenner and DeLamater, 2016), especially in older adults given elevated issues with impaired recall and biases like social desirability bias (Rabbitt et al., 1995; Wild et al., 2016). Since aging may impact the outcomes of self-report measures, more work needs to explore alternative report styles to ensure researchers are capturing actual changes caused by aging, rather than mere changes in self-report responses (Knäuper et al., 2016). Personality (Vazire, 2006), clinical (De Los Reyes et al., 2013), and cognitive (Pérez-Blanco et al., 2022) psychology researchers have implemented a promising alternative report style: informant reports. Informant reports provide information about a participant from someone close to them. This reporting style has shown valuable results, especially regarding cognition; meta-analyses have shown that, in healthy older adults, informant-reported cognitive complaints are more strongly associated with risk of developing mild cognitive impairment compared to self-report (Pérez-Blanco et al., 2022), and one study has shown that informant reports are more strongly associated with markers of Alzheimer’s disease than self-report (Rueda et al., 2015).

Although informant reports have shown utility in cognitive contexts (e.g., cognitive decline and Alzheimer’s progression), informant reports are underutilized in non-cognitive outcomes relevant to older adults, such as social and/or behavioral outcomes. However, some findings suggest they may be valuable in this context, particularly in measuring life satisfaction and daily functioning, key outcomes that reflect quality of life in aging populations (Rubenstein et al., 1984; Bravell et al., 2011; Siordia, 2012; Luhmann et al., 2016; Kim et al., 2020). However, many of these studies have the goal of assessing concurrence between self- and informant-reports at a single moment in time; the empirical literature has comparatively few examples exploring the reliability of informant-reported social and behavioral measures compared to self-report measures longitudinally following an experimental intervention protocol. Since daily functioning, life satisfaction, and quality of life are associated in older populations (Liao et al., 2024), properly assessing these outcomes is critical in monitoring quality of life changes in aging populations across an experimental intervention. Given the validity of informant-reported cognitive measures, and the potential value of informant-reported measures of life satisfaction and daily functioning, informant reports could provide additional validation of these changes.

The present study addresses this gap by comparing informant and self-reports of life satisfaction, financial capacity, and daily functioning within a randomized exercise intervention in older adults, a uniquely suitable context for this study given that exercise is an important public health focus in older populations. Indeed, reviews and meta-analyses show that physical activity contributes to health, wellbeing, and quality of life in older adults (Svantesson et al., 2015; Chen et al., 2020; Cunningham et al., 2020; García-Hermoso et al., 2020; Marquez et al., 2020) and has been shown to postpone cognitive decline (Iso-Markku et al., 2024). Additionally, the larger intervention study from which the data for this manuscript were extracted focused on a range of outcomes encompassing multiple domains thought to be potentially impacted by increased levels of physical activity and relevant to healthy aging (Parra-Rizo, 2017). These included social functioning, emotional functioning, and financial functioning. Findings demonstrated general improvements on several self-report measures including these outcomes (Gust et al., 2025), but existing analyses did not examine informant-report measures in these domains. Given these observed changes, this intervention provides a particularly suitable opportunity to explore discrepancies in self-report and informant-report measures across an experimental intervention.

To explore this, we utilized an Actor-Partner Interdependence Model (APIM) (Cook and Kenny, 2005), a method for dyadic analyses in which there is dependence between the assessments of the actor and the partner, as is the case when both participants and informants are reporting on the behavior of a single focal person, through self-report and informant reports, respectively. Though some, albeit limited, research has implemented the APIM technique for the analysis of informant reports in a non-experimental context (van Dulmen and Goncy, 2010), to our knowledge, this is the first analysis using the APIM technique on self-report and informant-report data in the context of an experimental intervention, and is the first analysis that uses APIM techniques to specifically analyze self-report and informant-report data in older adults. Given the value of APIM techniques in analyzing dyadic data, we hypothesized that the APIM technique would effectively capture concordances and discrepancies between self-report and informant-report measures across the experimental exercise intervention. In sum, the goal of this paper was to explore the relationship between self-report and informant-report measures of life satisfaction, financial capacity, and problems with daily functioning utilizing APIM. Through this, we aimed to provide more insight into reliable measures of behavioral outcomes especially relevant within aging populations.

## Materials and methods

### Participants

This study was approved by the University of Colorado Institutional Review Board (Protocol Number: 13-0392) and conducted in accordance with the Declaration of Helsinki. The data came from a randomized controlled trial testing whether exercise of different intensities could improve daily functioning, life satisfaction, and financial functioning in older adults and whether those improvements are related to variations in neurocognitive structure and function (Clinicaltrials.gov NCT02068612). Participants were recruited from the Denver/Boulder area of Colorado between April 2014 and November 2018 using numerous recruitment methods, including Craigslist, social media advertisements, ResearchMatch, university listservs, mailed flyers, and flyers posted in local community spaces. Participants were compensated up to $300 for participation in all study components. Eligibility screening was completed via telephone by research staff. Eligibility criteria required that participants were 60+ years old, not sufficiently physically active, and cognitively healthy. Additional details on the inclusion and exclusion criteria and methods can be found in previous publications (Karoly et al., 2021; Martin-Willett et al., 2021; Gust et al., 2025).

Informant reports were sourced from someone who knew the primary participant well (a spouse, child, family member or close friend) and was 18 years or older at the time of enrollment. Informants were required to know the participant well, given previous work suggesting that closeness between informants and participants may improve informant-report accuracy (An, 2022). Informant surveys were administered via telephone. Informants did not receive any additional compensation, and a participant’s compensation was not tied to the informant’s participation in the surveys. The study and all materials were approved by the University of Colorado Institutional Review Board.

### Design

A longitudinal randomized trial with informant-participant dyads was employed. Participants completed two baseline appointments followed by 16 weeks of the exercise intervention and a final follow-up appointment while informants completed two telephone surveys, one at the beginning of their respective participant’s exercise program and the other afterward (see below for further details).

### Procedure

#### Baseline appointments

At baseline, participants (*N* = 273) provided written informed consent (which included consent for contacting a provided informant) and completed a baseline health assessment and physician-supervised graded exercise test. If the participant was determined safe to participate in a VO_2_ peak test, they were asked to come back to a second appointment to complete this and answer self-report questions on their life satisfaction, financial capacity, and problems with daily functioning (*N* = 212). The two appointments were, on average, 18 days apart. Informant baseline surveys were administered after the first baseline appointment (*N* = 117).

#### Exercise intervention

Participants (*N* = 197) were randomly assigned to a low-intensity continuous training (LICT) condition (*N* = 101) or a moderate intensity continuous training plus high intensity interval (MICT + IT) condition (*N* = 96), based on previous literature on exercise interventions (de Souza et al., 2007). Participants then completed a 16-week supervised exercise intervention (at their assigned intensity/condition) three times per week at an exercise laboratory at the University of Colorado Boulder. Exercise sessions were supervised by trained research staff and lasted approximately 30 min. The majority of participants completed at least 90% (43/48) of the exercise sessions (78%, *N* = 154) while only 7% (*N* = 14) completed less than 25% (12/48) of exercise sessions. Previous analyses found effects of condition on self-report measures of loneliness only, so effects of condition were not expected for the measures analyzed in this manuscript and thus conditions were combined for this analysis (Gust et al., 2025).

#### Follow-up

After completing the 16-week exercise program, participants (*N* = 160) were asked the same self-report questions on life satisfaction, financial capacity, and problems with daily functioning. Informant surveys (*N* = 84) were likewise administered again after full completion of the exercise program.

### Measures

#### Life satisfaction

Life satisfaction was assessed using the five-item Satisfaction with Life Scale (Diener et al., 1985). Participants were asked how much they agree or disagree with each item (e.g., “So far I have gotten the important things I want in life”) on a seven-point scale ranging from 7 (strongly agree) to 1 (strongly disagree). Scores were averaged to produce a total self-report life satisfaction value, with higher scores indicating higher life satisfaction (α = 0.89). For informants, life satisfaction was assessed using the same scale in a third person format (e.g., “So far they have gotten the important things they want in life”), with the same response options. These items were likewise averaged to create a composite score, with higher scores indicating higher life satisfaction (α = 0.83).

#### Financial capacity

Financial capacity was assessed using a version of the financial capacity instrument (Marson et al., 2000). The measure included a five-item scale asking if participants had completed actions in the last 30 days associated with financial capacity (e.g., “In the last 30 days, did you pay your own bills such as rent, utilities, phone and transportation?”), with potential responses of “Yes,” coded as 1, “No,” coded as 0, or “Not Apply,” coded as missing. Recoded scores were averaged to generate a total self-report financial capacity value, with higher scores indicating higher financial capacity (α = 0.46). Informants were asked the same five items in reference to the participant’s financial actions in the last 30 days [e.g., Paid bills such as rent, utilities, phone and transportation (without prompting)]. Responses were on a five-point scale ranging from 1 (always) to 5 (never), with the option to respond, “No opportunity” or “Don’t Know.” To ensure comparability across self and informant reports and thus aid in data interpretation, informant responses were recoded to fit into the binary categories of the self-report version of the instrument; “never” was recoded to 0 (to match “No” in self-report) and any response ranging from “always” to “sometimes” was recoded to 1 (to match “Yes” in self-report). Answers of “No opportunity” or “Don’t know” were coded as missing. Like self-report, scores were averaged to generate a total informant-report financial capacity value, with higher recoded informant-report indicating higher financial capacity (α = 0.42). Given the low reliability of the financial capacity instruments, there are limits in the interpretability of the financial capacity results (see section “Discussion”).

#### Problems with daily functioning

A modified scale from the Health of the Nation Outcome Scales 65+ (Burns et al., 1999) including four items assessed problems with daily functioning. Participants were asked to rate their problems in the past 2 weeks with each item (e.g., “Problems with relationships”) on a five-point scale ranging from 0 (no problems) to 4 (very severe problems). Scores were averaged to generate a composite self-report daily functioning value, with higher scores indicating higher overall functioning problems (α = 0.73). For informants, daily functioning was assessed on the same scale in a third person format, with informants asked to rate the severity of problems that the participant had on each item (e.g., “Problems with the activities of daily living”) over the last 2 weeks. As with participants, scores were averaged to generate a composite value, with higher scores indicating more severe problems with functioning (α = 0.70).

### Statistical analysis

Primary analyses were conducted to explore differences in informant and participant ratings of participant life satisfaction, financial capacity, and problems with daily functioning via an APIM approach. APIM is a dyadic analytic framework designed to account for the statistical interdependence between members of a dyad while simultaneously estimating how an individual’s prior reports relate both to their own later outcomes (actor effect) and to their partner’s later outcomes (partner effect) (Cook and Kenny, 2005). This approach was particularly appropriate for the present study because participant and informant reports were inherently non-independent, as both members of the dyad were reporting on the same focal participant. Unlike approaches that analyze participant and informant reports separately, APIM allows the simultaneous estimation of concordance and divergence between reporting sources while accounting for dyadic interdependence.

Analyses were conducted in R version 4.3.2^1^ using the nlme package version 3.1-162 (Pinheiro et al., 2020) to estimate a multilevel APIM. Specifically, we ran three separate multilevel models for the three different outcomes. In each model, repeated observations were nested within individuals, who were themselves nested within participant-informant dyads. Fixed effects included time (baseline vs. follow-up), group (informant vs. participant), actor (within-person effects) and partner (cross-person effects) main effects, as well as interactions between group and each predictor. For example, actor effects estimated whether a participant’s baseline self-report predicted their own follow-up report, whereas partner effects estimated whether an informant’s baseline report predicted the participant’s follow-up self-report.

To estimate within- and cross-person effects, the actor (i.e., one’s baseline report) and partner (i.e., one’s partner’s baseline report) predictors were lagged predictors set to zero at the baseline occasion and assigned each person’s (or their partner’s) actual baseline report at follow-up, so that the follow-up outcome could be regressed directly on the prior (lagged) report. By interacting each of these with group (informant vs. participant), we obtained separate actor and partner slopes for participants and informants, allowing us to examine if actor and partner effects differed by role within the dyad. Where significant interaction effects were found, *post hoc* analyses using the emmeans package (Lenth, 2024) and graphs using the ggplot2 package (Wickham, 2016) were used to aid in interpretation. To account for non-independence inherent in dyadic data, we specified random intercepts for each person nested within dyad, thereby capturing both between-dyad variability and between-person variability within dyads. We also specified a compound symmetry structure to allow each person’s errors at baseline and follow-up to covary and allowing the residual variances to differ between participants and informants to address any role-specific heterogeneity. Models were estimated using restricted maximum likelihood (REML) and observations with missing values on variables included in a given model were excluded from that model using listwise deletion. Statistical significance was evaluated at α = 0.05.

## Results

### Descriptives

The majority of participants were female (61.17%) and White (91.57%) and on average around 68 years old (SD = 5.83). The remainder were Asian (2.93%), American Indian/Alaska Native (0.37%), and mixed race (1.83%) while 2.56% did not report their race. Informant demographic information was not assessed, though some information regarding the relationship to the participant was. The majority of informants reported being a romantic partner (e.g., spouse or simply “partner,” 53.85%) followed by being a family member (i.e., sibling, offspring, or parent, 32.48%) and being a friend (12.82%) while one person reported being the participant’s supervisor. Additionally, informants had known their participant partner on average 33.40 years, and the majority saw their participant partner (89.74%) and/or spoke with them by phone (94.87%) at least once a month. At baseline, both participants and informants reported generally high levels of life satisfaction (*M*s = 5.11 for both, ranges = 1.6–7 and 2–7, SDs = 1.26 and 1.14, respectively), low problems with daily functioning (*M*s = 0.42 and 0.65, ranges = 0–3.5 and 0–2.25, SDs = 0.54 and 0.62, respectively), and high financial literacy (*M*s = 0.85 and 0.98, ranges = 0–1 and 0.4–1, SDs = 0.20 and 0.10, respectively).

### Life satisfaction

The model predicting participant life satisfaction resulted in a main effect of actor just above the conventional threshold of significance and a significant main effect of partner, which was qualified by an interaction just above conventional significance thresholds between group and partner (see Table 1). The main effect of actor indicated a negative association between one’s baseline and follow-up scores [i.e., worse life satisfaction at baseline was associated better life satisfaction at follow-up; B = −0.12, standard error (SE) = 0.07] while the main effect of partner indicated that one’s partner’s baseline report was positively associated with one’s own follow-up reports (i.e., higher informant baseline reports of participant life satisfaction were related to better participant self-reported life satisfaction ratings at follow-up; B = 0.17, SE = 0.07). *Post hoc* analyses of the interaction indicated that there was a positive association between informant baseline reports and participant follow-up reports (B = 0.17, SE = 0.07, *p* = 0.01) but no association between participant baseline reports and informant follow-up reports (B = 0.01, SE = 0.07, *p* = 0.83).

**TABLE 1:** Mixed effect model findings across the three outcomes.

Variables	Life satisfaction			Financial capacity			Problems with daily functioning		
	B	SE	*P-*value	B	SE	*P-*value	B	SE	*P-*value
Time	−0.13	0.44	0.78	**−0.55**	**0.19**	**<0.01**	0.01	0.06	0.86
Group	−0.05	0.12	0.68	**0.13**	**0.02**	**<0.001**	**0.24**	**0.06**	**<0.001**
Actor	−**0.12**	**0.07**	**0.07**	−0.04	0.07	0.54	−**0.19**	**0.08**	**0.02**
Partner	**0.17**	**0.07**	**0.01**	**0.61**	**0.19**	**0.002**	0.00	0.06	0.98
Time × group	0.19	0.58	0.74	**1.10**	**0.20**	**<0.001**	−0.11	0.10	0.28
Group × actor	0.12	0.09	0.19	−**0.48**	**0.09**	**<0.001**	**0.28**	**0.12**	**0.02**
Group × partner	−**0.15**	**0.09**	**0.10**	−**0.63**	**0.19**	**0.001**	−**0.25**	**0.14**	**0.09**

Statistically significant and marginal results are bolded.

### Financial capacity

The model predicting financial capacity resulted in main effects of time, group, and partner qualified by time by group, group by partner, and group by actor interactions (see Table 1). In the case of the time by group interaction, which estimates mean-level changes across all individuals in the sample, *post hoc* analyses suggested that on average, participants’ reports indicated significantly decreased financial capacity over time (B = −0.55, SE = 0.19, *p* = 0.004), while informants’ reports indicated that participants’ financial capacity significantly increased over time (B = 0.55, SE = 0.06, *p* < 0.001).

*Post hoc* analyses probing the group by actor interaction, which estimates the rate of change in the outcomes within each person, indicated a significant negative association between informant baseline reports and their follow-up reports (i.e., a one-unit increase in an informant’s baseline rating of the participant’s financial capacity was associated with an 0.52-unit decrease in their financial capacity ratings of the participant at follow-up, *p* < 0.001). There was no significant association (*p* = 0.54) between participant baseline reports and their own follow-up reports.

*Post hoc* analyses examining the group by partner interaction showed a positive association between informant baseline reports and participant follow-up reports (B = 0.23, SE = 0.19, *p* = 0.002). Specifically, a one-unit increase in informants’ report of a participant’s baseline financial capacity was associated with a 0.61-unit increase in participants’ follow-up report. In contrast, there was no significant association between participant baseline reports and informant follow-up reports (B = −0.03, SE = 0.03, *p* = 0.34). The interpretation of these results is limited by the low reliability of the financial capacity instruments; a more detailed analysis of the reliability concerns appears in the discussion.

### Problems with daily functioning

The model predicting problems with daily functioning resulted in similar main effects as the financial capacity model. There were main effects of group and actor which were qualified by a significant interaction between group and actor and an interaction just above the conventional threshold for significance for group and partner (see Table 1). *Post hoc* analyses of the group by actor interaction suggested a significant negative association between participant baseline reports and participant follow-up reports (B = −0.19, SE = 0.08, *p* = 0.02; i.e., a one-unit increase in a participant’s own baseline report of problems with daily functioning was associated with a 0.19-unit decrease in their own follow-up report). There was no significant association between informant baseline reports and informant follow-up reports (B = 0.09, SE = 0.09, *p* = 0.31).

For the group by partner interaction, *post-hoc* tests indicated a negative association between participant baseline reports and informant follow-up reports (B = −0.25, SE = 0.13, *p* = 0.06). Specifically, a one-unit increase in participants’ baseline self-rated problems with daily functioning was associated with a 0.25-unit decrease in informants’ rating of the participant’s daily functioning at follow-up. In contrast, there was no significant association between informant baseline reports and participant follow-up reports (B = 0.00, SE = 0.06, *p* = 0.98).

## Discussion

This dyadic analysis aimed to understand self-report and informant-report measures of life satisfaction, financial capacity, and daily functioning in older adults in the context of a structured exercise intervention. While prior work has largely emphasized informant reports in the context of cognitive decline and neurodegenerative disease progression (Rueda et al., 2015; Pérez-Blanco et al., 2022), our findings suggest that informant perspectives also hold unique value for non-cognitive outcomes.

Across measures, the relationship between self-reported and informant-reported outcomes varied. For life satisfaction, the main effect of partner and *post hoc* analyses suggest that higher informant-reported baseline life satisfaction (i.e., the informant’s opinion of the participant’s life satisfaction at baseline) was associated with higher participant self-reported life satisfaction (i.e., the participant’s own ratings of their life satisfaction) following the 16-week experimental protocol. Additionally, lower participant baseline self-reported life satisfaction was marginally associated with higher follow-up self-reported life satisfaction. For financial capacity, a pattern of divergence emerged. While self-reported financial capacity on average decreased, informant-reported financial capacity on average increased from baseline to follow-up, suggesting differences in how participants and informants perceived change over time. Higher informant baseline reports were also associated with lower informant follow-up reports but higher participant follow-up reports. In contrast, for problems with daily functioning, self-report and informant-report results were more concordant. Participants saw an overall decrease in problems with daily functioning from baseline to follow-up, and higher participant baseline reports of problems with daily functioning were associated with lower follow-up reports in both participants and informants.

Taken together, these findings suggest that convergence between self-report and informant-report outcomes may differ across domains of functioning. For life satisfaction, one possible interpretation is that informants’ impressions at baseline capture subtle aspects of the participants’ wellbeing that continue to shape their later self-perceptions. This aligns with broader work showing that close others can provide unique and valid information about a person’s subjective experience, sometimes noticing patterns that participants themselves may underreport or only recognize over time (Schinka, 2010; Scholz and Donders, 2024). In contrast, the divergence observed for financial capacity suggests that participants and informants may have evaluated financial functioning using different reference points or criteria over time. One possibility is that participants’ self-ratings reflected perceived autonomy or confidence in their abilities after engaging in the structured exercise intervention, whereas informants may have based their ratings more heavily on observable day-to-day financial skills. Prior work on multi-informant assessment similarly suggests that discrepancies between reporters are common and may reflect differences in perspective, access to observable behavior, or interpretation of the construct being assessed rather than simple measurement error alone (Schinka, 2010; De Los Reyes et al., 2013). At the same time, these interpretations should be considered alongside the low reliability of the harmonized financial capacity measure and the limitations introduced through recoding informant responses into binary categories (see the discussion on limitations below). Lastly, the relative convergence observed for problems with daily functioning suggests that changes in observable daily functioning may have been more consistently perceived across reporting styles. Across report styles, participants’ daily functioning seemed to improve throughout the experimental protocol. This also reinforces prior evidence (Karoly et al., 2021; Martin-Willett et al., 2021; Gust et al., 2025) that physical activity interventions can enhance functional ability in older adults and suggests that improvements are observable both to the participant themselves and to those who are close to them.

Collectively, these results show the value of utilizing APIM for modeling self-report and informant-report data simultaneously in older adults. Standard approaches that treat self- and informant reports independently may miss the opportunity to model their interdependence and reciprocal influence. In contrast, the APIM provides a framework to disentangle these influences, providing a clearer picture of both concordance and discrepancy between report styles. The present findings suggest that informant reports provide complementary, rather than interchangeable, information compared to the use of self-report alone.

From a practical perspective, these findings support the inclusion of informant-report measures in intervention research involving older adults, especially when outcomes may be vulnerable to self-report biases or when subtle behavioral changes may be more observable to close others. It is still unclear what causes the observed differences between participant and informant reports, and further research should examine the effect of differences in relationship type (e.g., parent and offspring vs. spouse vs. friend), context (i.e., do certain experimental intervention contexts improve convergence of self-report and informant-report measures over time?), and other factors that may influence outcomes by reporting style. Indeed, understanding how these factors may influence participant-informant reports may help to provide clearer guidelines in future studies including specific prescriptions of who should be included as an informant and what type of exercise interventions may be most useful to provide accurate data.

Some limitations of this study should be noted. First, the self-report and informant-report scales of financial capacity had low reliability, which constrains interpretability of the results for this measure. We believe that the low reliability may be a result of the binary recoding used in data analysis. To properly compare across report styles, informant-report financial capacity was recoded to the binary “Yes” vs. “No” used in self-report, such that only two responses were possible for both report styles. Therefore, it is possible that the results of the financial capacity analyses reflect measurement limitations rather than actual differences between report styles. We recommend that future work uses the five-point scale across report styles to help clarify whether the observed discrepancies reflect meaningful differences in perception or artifacts of measurement construction.

In terms of broader limitations, the relationships of participants and informants varied (e.g., spouses, parents/offsprings, friends), and differences by relationship type were not examined in this analysis but could meaningfully shape reporting patterns. For instance, spouses may be more attuned to participants’ daily functioning, whereas adult children may be more attuned to financial skills. Likewise, this study could not differentiate between co-residing and non-co-residing informants. Since the relationship between informants and participants, as well as co-residing status, may influence results, future work should include these variables in analyses. Finally, our sample was relatively demographically homogenous with 91.57% of participants identifying as white, and we did not collect detailed demographic information from informants. Further research replicating this analysis with more diverse and well-defined populations is recommended to better generalize these results to the broader population. Finally, all self-report measures are vulnerable to recall and social desirability bias.

APIM analyses for self-report and informant-report data are an underutilized tool for measuring experimental outcomes of interventions with older adults. These analyses can allow researchers to test outcome measures of informant reports, compare these results to self-report measures, and effectively manage the interdependence of self-report and informant-report data. By collecting both report styles and utilizing a dyadic analysis, more complete results can be collected than would be possible utilizing either reporting style alone. This suggests that informant reports are a valuable addition to experimental interventions in older adults, and APIM analyses are a powerful tool to most effectively interpret results when both informant-report and participant self-report data are collected.

## Data Availability

The raw data supporting the conclusions of this article will be made available by the authors, without undue reservation.

## Footnotes

1
https://posit.co/
